# Design and Evaluation of Hydrophobic Ion Paired Insulin Loaded Self Micro-Emulsifying Drug Delivery System for Oral Delivery

**DOI:** 10.3390/pharmaceutics15071973

**Published:** 2023-07-18

**Authors:** Jahanzeb Mudassir, Afsheen Raza, Mahtab Ahmad Khan, Huma Hameed, Gamal A. Shazly, Ali Irfan, Sadia Jafar Rana, Khizar Abbas, Muhammad Sohail Arshad, Sajjad Muhammad, Yousef A. Bin Jardan

**Affiliations:** 1 Faculty of Pharmacy, Bahauddin Zakariya University, Multan 60800, Pakistan; 2 Faculty of Pharmaceutical Sciences, University of Central Punjab (UCP), Lahore 54000, Pakistan; 3 Department of Pharmaceutics, College of Pharmacy, King Saud University, Riyadh 11451, Saudi Arabia; 4 Department of Chemistry, Government College University Faisalabad, Faisalabad 38000, Pakistan; raialiirfan@gmail.com; 5 Department of Neurosurgery, Medical Faculty, Heinrich Heine University, Moorenstrasse-5, 40225 Düsseldorf, Germany; 6 Department of Neurosurgery, University of Helsinki and Helsinki University Hospital, 00290 Helsinki, Finland

**Keywords:** oral drug delivery, insulin, SMEDDS, hypoglycemic effect, hydrophobic ion pair loaded, bioavailiability

## Abstract

Despite several novel and innovative approaches, clinical translation of oral insulin delivery into commercially viable treatment is still challenging due to its poor absorption and rapid degradation in GIT. Thus, an insulin-SDS hydrophobic ion pair loaded self-microemulsifying drug delivery system (SMEDDS) was formulated to exploit the hypoglycemic effects of orally delivered insulin. Insulin was initially hydrophobically ion paired with sodium dodecyl sulphate (SDS) to enhance its lipophilicity. The successful complexation of Insulin-SDS was confirmed by FTIR and surface morphology was evaluated using SEM. Stability of insulin after its release from HIP complex was evaluated using SDS PAGE. Subsequently, Ins-SDS loaded SMEDDS was optimized using two factorial designs. In vitro stability of insulin entrapped in optimized SMEDDS against proteolytic degradation was also assessed. Further, antidiabetic activity of optimized Ins-SDS loaded SMEDDS was evaluated in diabetic rats. Insulin complexed with SDS at 6:1 (SDS/insulin) molar ratio with almost five-fold increased lipophilicity. The SMEDDS was optimized at 10% Labraphil M2125 CS, 70% Cremophore EL, and 20% Transcutol HP with better proteolytic stability and oral antidiabetic activity. An Ins-SDS loaded SMEDDS was successfully optimized. Compared with insulin and Ins-SDS complex, the optimized SMEDDS displayed considerable resistance to GI enzymes. Thus, the SMEDDS showed potential for effective delivery of macromolecular drugs with improved oral bioavailability.

## 1. Introduction

The advancement in the field of pharmaceutical technology has focused on the need to develop delivery system for targeted delivery of peptides and protein drugs. Among these self-microemulsifying drug delivery system (SMEDDS) is a superior choice for delivery of macromolecular drugs in multiple scenarios. It not only offers improved oral bioavailability of drugs by allowing efficient drug transport across absorptive membranes but also shields encapsulated drugs against severe enzymatic hydrolysis in GIT [1]. Moreover, simple manufacturing processes without consumption of any external heating or energy aid also favors delivery of heat labile proteins and peptides through the system. However, hydrophilic nature of proteins makes it almost impossible to dissolve in lipophilic oil phase of system. To deal with the issue, hydrophobic ion pair technique (HIP) was introduced by researchers that enhance the lipophilic nature of proteins and peptides simply by incorporating a hydrophobic chain in the structure [2]. The technique involves complexation of proteins’ ionizable groups with counterions of complex forming agents such as amphiphilic molecules or surfactants at a particular pH [3]. Complex is usually formed between basic amino acids of proteins primarily lysines and arginines and anionic surfactants. In the presence of excess of oppositely charged ions with higher affinity, complex dissociates into its components [4]. Similar phenomena were used to encapsulate insulin in SMEDDS.

In this present study, insulin was initially hydrophobically ion paired with sodium dodecyl sulphate (SDS) (referred as hydrophobic ion pair HIP) to enhance its lipophilicity. The successful complexation of Insulin-SDS leads to its further loading in self microemulsifying drug delivery system. Hydrophobic Ion pair (HIP) formation was confirmed by FTIR. Stability of insulin in HIP complex was a major concern and was evaluated using SDS-PAGE. In vitro stability of optimized SMEDDS against proteolytic degradation was also assessed. Further, anti-diabetic activity of optimized Ins-SDS loaded SMEDDS was evaluated in diabetic rats.

## 2. Materials and Methods

### 2.1. Materials

Human insulin was purchased from Novo Nordisk(Bagsvaerd, Denmark). Sodium dodecyl sulphate (SDS) was supplied by Duksan Pure Chemicals Korea (Duksan Pure Chemicals Co., Ltd., Ansan, Korea). Labrafil M2125 CS and Transcutol HP were purchased from Gattefose Lyon France. Cremophore EL was bought from Sigma Aldrich (St Louis, MO, USA). Trypsin and Chymotrypsin were also supplied by Sigma Aldrich. Tris buffer and Laemelli buffer were also provided by Sigma Aldrich. Streptozotocin was purchased from Otto Chemie pvt. Ltd. (Mumbai, Maharashtra). All other chemicals and reagents used were of analytical grade.

### 2.2. Methods (Preparation of Ins-SDS HIP Complex)

Acidified solution (pH 2.5) of SDS and insulin were prepared independently. SDS solution was added dropwise in insulin solution in different molar ratios, i.e., 3:1, 6:1, and 9:1 of SDS/insulin in separate vessels [5]. The resultant white precipitates indicated HIP formation. Complexes were recovered by centrifugation of cloudy solution at 5000 rpm for 6 min. The obtained precipitates were washed and lyophilized. The lyophilization was performed in the presence of trehalose as cryoprotectant. The specified amount of formulation was mixed with trehalose and then frozen overnight at −20 °C prior to lyophilization. The frozen sample was then lyophilized using freeze drying system (Labconco Corporation, Kansas City, MO, USA) for 24 h to allow complete drying of samples.

#### Optimization of HIP Complex

Optimization of HIP complex was conducted on the basis of complexation efficiency. Thus, insulin concentration in supernatant was analyzed spectrophotometrically at λmax 270 nm (UV-vis spectrophotometer, Hitachi) as a reflection of unbound insulin concentration and used for the estimation of optimum insulin/SDS molar ratio by using the following equation:

(1) $$\mathrm{Percentage}\;\mathrm{Complexation}\;\mathrm{Efficiency}=\frac{I_{T}-I_{F}}{I_{T}}\times 100$$

where $I_{T}$ is the amount of insulin initially added and $I_{F}$ is the amount of insulin present in the supernatant.

### 2.3. Characterization of Ins-SDS Complex

#### 2.3.1. Determination of Log P and Apparent Insulin Solubility

Acidified solutions (pH 2.5) of insulin and SDS, each 1 mg/mL, were prepared independently. An amount of 0.6 mL of SDS solution was added dropwise into 0.6 mL of insulin solution followed by the addition of 1.2 mL of 1-octanol. After 4 h of magnetic stirring, the mixture was centrifuged for 30 min at 5000 rpm. Insulin concentration in both phases was measured spectrophotometrically at 270 nm.

(2) $$\mathrm{Partition}\;\mathrm{Coefficient}=\frac{Insulin\;Concentration\;in\;1-Octanol}{Insulin\;Concentration\;in\;Water}$$

For determination of insulin solubility, the increasing amount of insulin was added in purified water and stirred at 25 ± 2 °C for 12 h. The apparent aqueous solubility was determined considering the minimum amount of purified water which gave a transparent solution when visually evaluated. 

#### 2.3.2. FTIR

FTIR was performed to analyze the possible interaction between insulin and SDS. Thus, insulin, SDS, and Ins-SDS complex were scanned across the range of 4000–397 ${nm}^{-1}$ using Thermo scientific, Waltham, MA, USA.

#### 2.3.3. SEM

Scanning electron microscope (Evo LS 10 Zeiss, Oberkochen, Germany) was used to visualize the morphological features of lyophilized HIP complex. The samples were fastened on a brass stub with the help of adhesive tape and a small layer of gold was applied to make them electrically conductive. SEM images were captured at 20 kv accelerating voltage from different angles at 100×–1000× magnification values.

#### 2.3.4. Sodium Dodecyl Sulphate Polyacrylamide Gel Electrophoresis (SDS-PAGE)

Structural integrity of insulin in HIP complex was investigated using SDS-PAGE as described earlier [6]. Briefly, insulin sample and molecular weight markers were boiled for 5 min after mixing with 2-mercaptoethanol and Laemelli’s buffer in separate micro-centrifuge tubes. The electrode assembly containing gel cassette was fixed in clamp stand. The wells of the gel were filled with electrophoresis buffer, which was poured into the casting frame’s entrance. In the well, 20 µL of denatured sample was added. After around 45 min, the gel was running at 60 V when the running front hit the gel’s bottom. Silver staining was used for visualization of protein.

### 2.4. In Vitro Studies

#### Dissociation of HIP

Briefly, 2 mg of Ins-SDS was incubated independently at 37 °C in 1 mL of distilled water (DW), 0.01 M HCl (pH 2), and 10 mM phosphate buffer (PB) solution (pH 6, 6.8 and 7.4). All of these media were initially dispersed with 137 mM NaCl. Additionally, HIP was incubated in PB (10 mM, pH 7.4) with varying ionic strength (10, 100 and 150 mM). Samples were centrifuged at 10,000 rpm for 10 min at predetermined time intervals [7]. Subsequently, an aliquot of 100 µL were withdrawn and analyzed spectrophotometrically after required dilution.

### 2.5. Construction of Pseudo-Ternary Phase Diagram

Water titration method was employed for the development of ternary phase diagram of oil, surfactant, and co-surfactant. Labraphil M2125 CS, Cremophore EL, and Transcutol HP were selected as oil, surfactant, and cosurfactant. Surfactant and cosurfactant (Smix) were mixed in a specified manner (1:1, 1:2, 1:3, and 1:4). Afterwards, the lipophilic phase and Smix were taken in specific weight ratios of 1:9, 2:8, 3:7, 4:6, 5:5, 6:4, 7:3, 8:2, and 9:1 [8]. The resultant emulsions were visually evaluated for any turbidity or precipitation.

### 2.6. Experimental Optimization of HIP-SMEDDS

Scheffe’s mixture design was used to optimize composition of SMEDDS as shown in Table 1. It is anticipated to fit response surface methodology in order to optimize intricate formulation designs. Experimental model designing and statistical analysis were accomplished using Design Expert 13. The contents of Labraphil M2125 CS (X_1, *w*/*w*%), Cremophore EL (X_2, *w*/*w*%), and Transcutol HP (X_3, *w*/*w*%) were adjusted within ranges of 10–30%, 30–70%, and 10–50%, respectively. The drug loading of HIP in SMEDDS (Y_1), droplet size (Y_2), emulsification time (Y_3), and transmittance (Y_4) were various dependent variables evaluated. Regular two-level factorial design was utilized for estimating responses. The eight designed experiments were fitted to various polynomial models [9]. Appropriateness of each fitted model for independent variables was confirmed by comparing numerous fit statistics provided by ANOVAs. The optimization was performed using desirability function.

#### 2.6.1. Drug Loading ($Y_{1}$)

An excess of insulin-SDS HIP complex was added in a glass vial containing 1 g of SMEDDS. The mixture was vortexed for 20 min and centrifuged for 45 min at 5000 rpm. Later on, the drug loading was measured.

#### 2.6.2. Droplet Dimensions ($Y_{2}$)

A reconstituted microemulsion was prepared by mixing 100 mg of SMEDDS with 10 mL of distilled water. The sample preparation was incubated at room temperature for 30 min and then subjected to size measurement, the droplet sizes of SMEDDS were determined by dynamic light scattering using a particle size analyzer (Zetasizer 1000HSA Malvern Instruments, Malvern, UK).

#### 2.6.3. Self-Emulsification Time ($Y_{3}$)

An amount of 1 g of each test formulation was added to 250 mL of aqueous medium with magnetic stirring at 100 rpm at room temperature, 37 °C ± 2 °C. Time required by each formulation to form a homogenous dispersion was noted.

#### 2.6.4. Transmittance ($Y_{4}$)

The percentage transmittance of sample formulations were measured at 276 nm using UV spectrophotometer. Microemulsions were prepared by adding 100 mg of each sample formulation in 10 mL of purified water and transmittance was calculated by using the following equation:

(3) $$\mathrm{Percentage}\;\mathrm{Transmittance}=100\;\times \;10^{-A}$$

### 2.7. Enzymatic Degradation Studies

#### 2.7.1. Stability Assessment against Proteolytic Degradation

Stability of Ins-SDS loaded optimized SMEDDS against proteolytic degradation was investigated using simulated intestinal fluid (SIF) [10]. To prepare SIF, trypsin and chymotrypsin were added to the Tris buffer. All the samples were adjusted to final insulin conc. of 0.2 mg/mL. Insulin and Ins-SDS samples were prepared by dissolving them independently in 0.05 M PB (pH 7) with Tween 20 (0.05%). Afterwards, 250 µL of SIF was added to each sample preparation and subjected to mild shaking (100 rpm) at 37 °C. Later, the degradation reaction was inhibited by the addition of 500 mL of 0.1% TFA in methanol at scheduled time points up to 4 h. The concentration of remaining insulin in each sample was quantified using HPLC. 

#### 2.7.2. Quantification by Using HPLC

An acidic mobile phase was used for detection of insulin. The mobile phase consisted of 0.2 M sodium sulphate anhydrous (adjusted to pH 2.3 with ortho phosphoric acid) and acetonitrile (74:26). The aqueous solution was filtered through 0.45 µm pore size nylon membrane filter (Whatman international, Maidstone, UK) under vacuum and degassed prior to use. The analysis was run at a flow rate of 1.2 mL/min and sample injection volume of 20 µL. The detector was set a wavelength of 214 nm. The chromatographic analysis was performed using a Shimadzu Prominence HPLC system (Kyoto, Japan) consisting of an in-line DGU-20A3 Prominence degasser, LC-20AD Prominence solvent delivery pump, SIL-20A HT prominence auto sampler, CTO-10AS VP column oven, and SPD-M20A Prominence UV-VIS detector. Data acquisition and analysis were performed using Shimadzu LC solution software Version 1.24 SP1 (Kyoto, Japan).

### 2.8. In Vivo Study

#### Antidiabetic Effect Study in Diabetic Rats

Glucose lowering effect of orally administered optimized SMEDDS was monitored in diabetic rats. Study was performed with the approval Pharmacy Ethical Committee, Bahauddin Zakariya University, Multan, Pakistan (249/PEC/2023 dated 25 March 2023). Diabetes was introduced in rats weighing 220–240 g by injecting streptozotocin solution at the dose of (45 mg/kg body weight) intraperitoneally. Fasting blood glucose (FBG) level of all the rats was measured after 3 days using glucometer (AccuChek Performa, Hoffman-La Roche, Basel, Switzerland). Rats with FBG level 200–250 mg/dL were considered diabetic and were included in the study. All rats were fasted over night before experiment and start of treatment. Diabetic rats were divided randomly in 5 groups with 5 rats per group. Each treatment group received orally administered PBS, insulin solution (Ins-PO), Ins-SDS solution per oral, optimized SMEDDS, insulin (Ins-SC) at dose of 5 IU/kg. All other preparations were also administered orally at a dose of insulin equivalent to 50 IU/kg body weight. Blood samples were collected at regular time intervals, i.e., 0, 0.5, 1, 1.5, 2, 3, 4, 6, and 8 h and measured for hypoglycemic effects using glucometer [6].

## 3. Results and Discussion

### 3.1. Preparation and Optimization of Ins-SDS HIP Complex

HIP of peptides with surfactants is an efficient tool to enhance its lipophilic characteristics. In this present study, insulin was hydrophobically ion paired with SDS at pH 2.5 which is less than isoelectric point of insulin, i.e., 5.35–5.45. Insulin is a polypeptide, having 51 amino acids with 6 acidic and 6 basic groups. At this pH, all the acidic groups (four Glutamate residues and two C-terminal carboxylates) of insulin had become unionized while basic groups received protonated. These six positively charged amino acids (two Histidine, one Arginine, one Lysine and two N-terminal amino acids) could form efficient bonds with negatively charged sulfate groups of SDS [11]. As SDS solution was added dropwise in insulin solution, the mixture became cloudy with the formation of precipitates which was an indication of a decrease in aqueous solubility due to enhanced lipophilic character [2]. However, stoichiometric molar ratio of 6:1 displayed maximum complexation efficiency with minimum aqueous solubility. Meanwhile, it was also observed that upon further addition of SDS in insulin solution, i.e., greater than 6:1 molar ratio, the turbidity was reduced and precipitates began to re-solubilize.

#### Mechanism of HIP Formation

The surfactant binds with peptide below critical micellar concentration where polar head groups of surfactants interact electrostatically with oppositely charged peptide side chains while subsequent hydrophobic interactions also occurred between alkyl chains and adjacent hydrophobic patches [12]. Thus, it was speculated that all insulin sites might became saturated with SDS at a stoichiometric molar ratio of 6:1 and upon further addition of SDS micelles were formed. The micelles re-solubilize efficiently and was further responsible for reduction of complexation efficiency. The results were found to be in concordance with previous studies [13].

### 3.2. Characterization of HIP Complexes

#### 3.2.1. Log P Determination

To confirm the enhanced lipophilic character of HIP, its partitioning in n-octanol/water system was determined. The solubility of free insulin in n-octanol was initially almost 0.017 mg/mL, which was an increase of up to 4.87 mg/mL when bound with SDS [14,15]. The partitioning of HIP in n-octanol improved almost about five-fold compared to unbound insulin. However, in this present study it was hypothesized that the optimized formulation does not possess excessive or free SDS. However, authors reported in a similar study that excessive or additional SDS available in formulation formed micelles. These SDS micelles are considered responsible to re-solubilized HIP [15]. These HIP-nanocomplexes developed via electrostatic and hydrophobic interactions. It is the strategy to from hydrophobic complexes from charged hydrophilic molecules, and based on the pH-related and stoichiometric replacement of polar counter-ions with an ionic surfactant [14].

Furthermore, insolubility of insulin in organic solvents is partly due to the significant charge that most proteins (insulin) possess at a given pH. On masking that charge, altered solubility characteristics of insulin are achieved. Moreover, in this present case, HIP complex was prepared at fixed pH, and the effect of pH of the aqueous phase on the solubility of insulin as well as on Ins-SDS complex is reported elsewhere [14,15].

#### 3.2.2. FTIR

**Figure 1:**
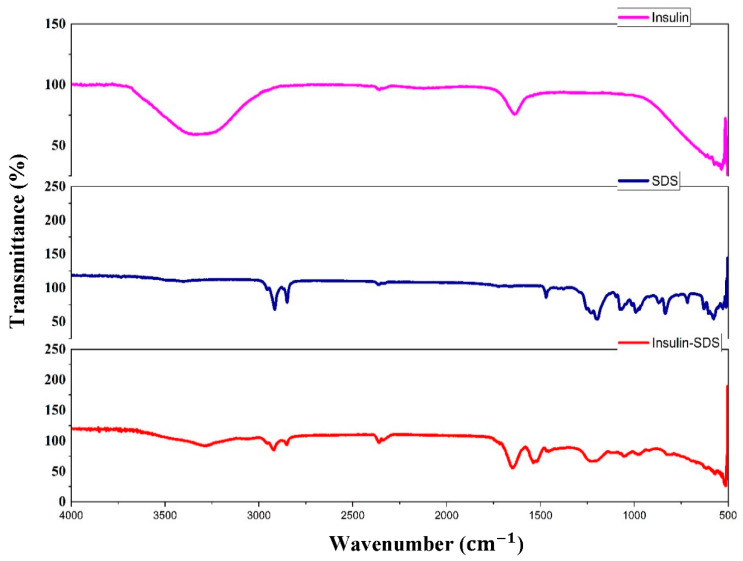
Fourier Transform Infrared Spectra of Insulin, SDS, and Insulin-SDS HIP complex.

Figure 1 illustrates infrared spectra of insulin, Ins-SDS complex, and SDS. Four distinctive peaks at 3299.60 (amide A), 1639.19 (amide I), 1542.77 (amide II), and 1232.29 (amide III) ${cm}^{-1}$ were observed in insulin spectrum. Similarly, four characteristics absorption bands at 2915.84 (C$H_{2}$ asymmetric vibrations), 2850.27 (C$H_{2}$ symmetric vibrations), 1211.07 (${SOO}^{-}$ asymmetric vibrations), and 1076.08 (${SOO}^{-}$ symmetric vibrations) ${cm}^{-1}$ were detected in SDS spectrum. Comparing the IR spectra of insulin-SDS complex with native SDS, the distinctive peak at 1211.07 ${cm}^{-1}$ corresponding to S-O-H stretching vibration mode was relatively less intense and shifted towards lower wavelength in the HIP spectra. These results suggest that interactions between the anionic sulphate of SDS and cationic amine of insulin might be responsible for successful ion pair formation.

#### 3.2.3. SEM

**Figure 2:**
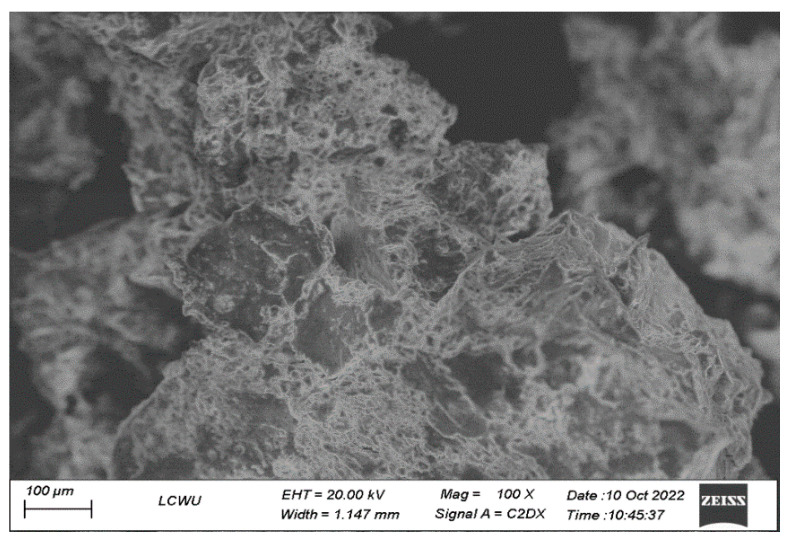
SEM images of Insulin-SDS complex.

The morphological characteristics of insulin-SDS complex were represented using SEM. An aggregated mass with a rough surface was observed in insulin-SDS (Figure 2) rather than defined insulin crystallinity. Such transformation of morphology indicated that the complex had successfully formed.

#### 3.2.4. Sodium Dodecyl Sulphate Polyacrylamide Gel Electrophoresis (SDS-PAGE)

**Figure 3:**
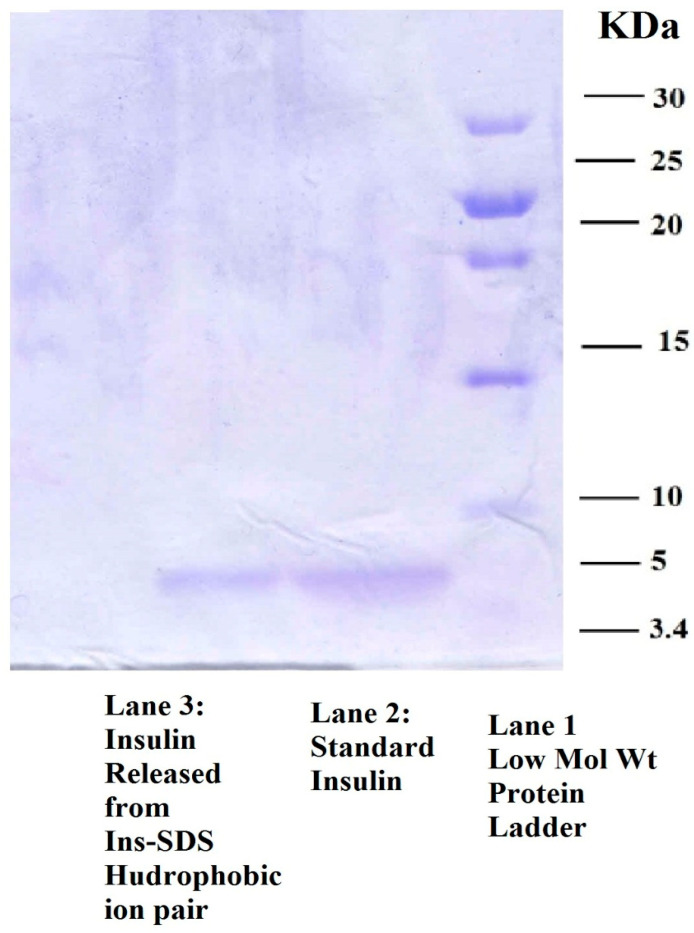
SDS-PAGE of insulin representing Standard Low Molar Weight Protein Ladder in Lane 1, Standard Insulin in Lane 2, and Insulin recovered from Ins-SDS HIP complex.

The experiment was performed to demonstrate the structural stability of insulin after hydrophobic ion pairing. Figure 3 illustrates a representative image of blue silver-stained gel with visible bands of insulin. Lane 1 shows standard bands for protein molecular weight marker. This shows a level to which high molecular weight protein, i.e., 30 kDa, travel to highest level while low molecular weight proteins remain at lower level. Lane 2 displays the insulin standard. Lane 3 represents insulin released from Ins-SDS HIP. The visible bands at approximately 5.4 kDa in Lane 2 and 3 points out structural similarity between both components as represented by each band. This study confirms that insulin does not undergo any structural deformation during complexation and decomplexation. Thus, there was no deleterious effect of ion pairing observed on insulin integrity [6].

### 3.3. In Vitro Dissociation Studies

#### 3.3.1. Dissociation Study of HIP

Dissociation studies of HIP were conducted to evaluate the influence of pH and ionic strength of release medium with respect to time on the de-complexation behavior of Ins-SDS complex.

##### Dissociation in DW with 0 Ionic Strength

**Figure 4:**
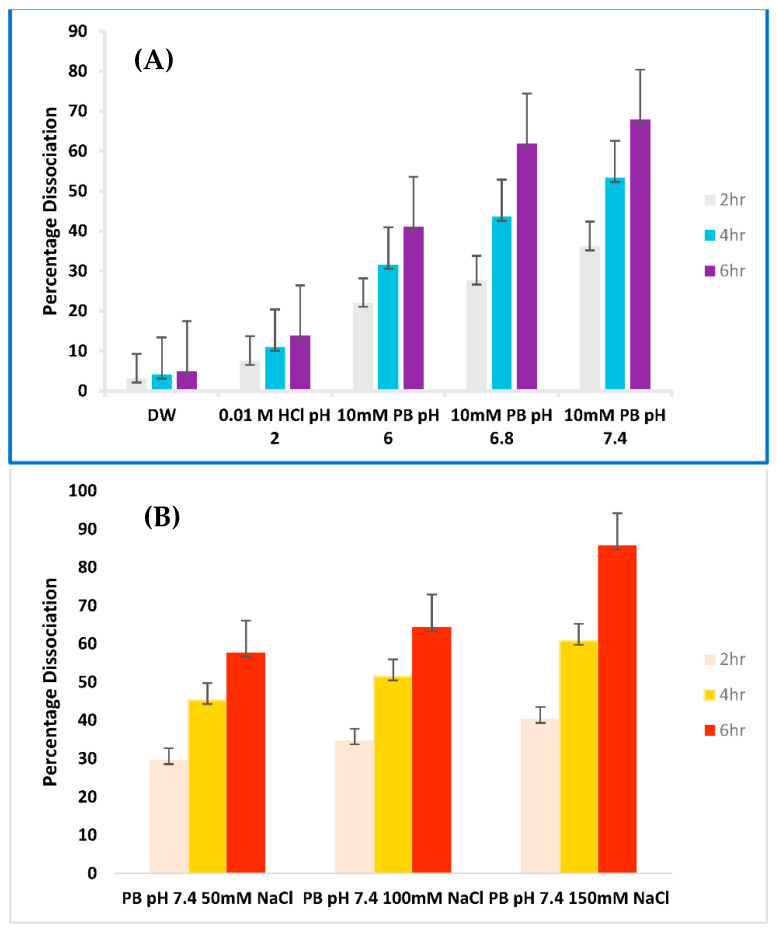
(**A**). Dissociation Profile of Ins-SDS Complex influenced by pH at 2 h (grey bars), 4 h (blue bars), and 6 h (purple bars) (**B**): Dissociation Profile of Ins-SDS Complex influenced by ionic strength 2 h (pink bars), 4 h (yellow bars), and 6 h (red bars).

In this study, incubation of HIP in DW served as a negative control. Figure 4A elucidates the influence of pH with respect to time on the dissociation behavior of Ins-SDS complex in different release medium. Only a negligible amount of complex, almost 5%, dissociated when incubated in DW. This confirms the stability of HIP in DW due to existence of ionic interactions between insulin and SDS along with hydrophobic interactions.

##### Effect of pH on Dissociation of HIP at 10 mM Ionic Strength

Meanwhile, a gradually increasing dissociation pattern was observed by increasing the pH of release medium. A dissociation of 13.85 at pH 2 (0.01 M HCl) and 67.85% at pH 7.4 (PB) was observed after 6 h. This shows that dissociation of HIP was promoted at alkaline pH. This might be due to pH driven charge negation of released insulin and SDS which prevents the re-complexation of released moieties that may occur at lower pH.

##### Effect of Increasing Ionic Strength at Constant pH

On the other hand, Figure 4B displays the effect of concentrations of ions over time on the dissociation profile of HIP. Ins-SDS exhibited higher complex dissociation when subsequently exposed to PB medium with higher ionic strength. This suggests that dissociation of HIP was promoted by increasing salt concentration. A total of 85.7% dissociation of Ins-SDS complex was observed at 150 mM NaCl concentration, similar to physiological conditions within 6 h. A similar dissociation pattern was observed for lysozyme-SDS complex in PB with increasing ionic strength (0.1 M, 0.5 M, 1 M NaCl) [16].

Such release pattern might be attributed to ionic dissociation of HIP followed by charge screening. HIP is stabilized by delicate ionic interactions and can be disassemble when exposed to higher ionic strength release medium. Dissociation results from salts in the release medium being able to access the complex and outcompete the HIP. Recomplexation is unlikely due to charge screening of regenerated species by high ionic strength of release medium. These findings are consistent with past research supporting that dissociation of HIP is promoted at higher pH and ionic strength over time [16]. Previous studies suggest that drug release from oil droplets is associated with the lipophilic character of HIP as well as ionic strength of release medium [17,18]. Conversely, complex in-vivo environment due to the presence of endogenous anions such as phosphatidylinositol, phosphatidylserine, and bile acids may alter the release behavior of HIP and should be further investigated.

### 3.4. Pseudo Ternary Phase Diagram

**Figure 5:**
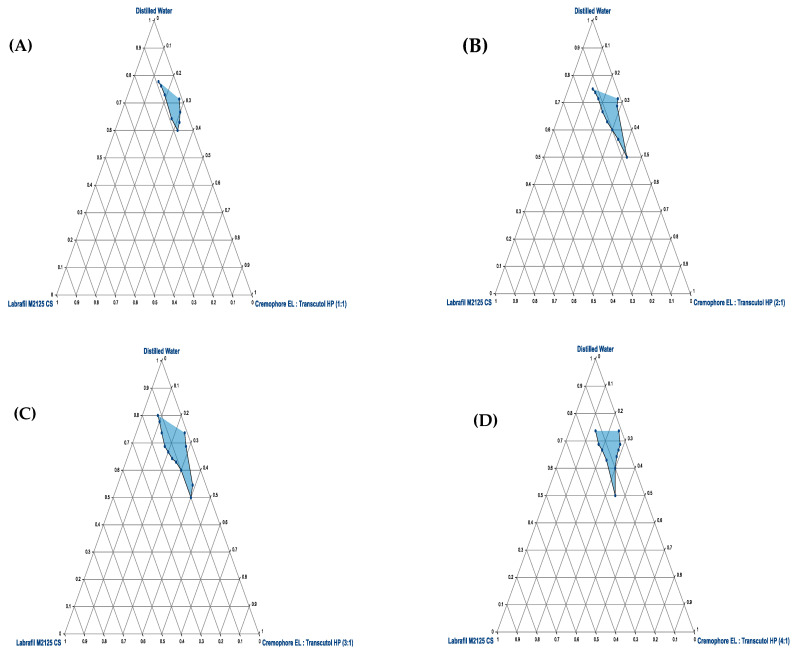
Pseudo Ternary Plots of Labrafil M2125 CS (oil), Cremophore EL (surfactant), and Transcutol HP (cosurfactant) representing MER (blue surface area) at different Smix ratios (**A**) 1:1 (**B**) 2:1 (**C**) 3:1 (**D**) 4:1.

To assess the microemulsion region, various mixtures of oil and Smix were evaluated in specific molar ratios using ternary diagram. It is mandatory to mention that the phase diagram was plotted in the absence of Ins-SDS ion pair complex. Each Smix ratio generated an independent ternary phase diagram. Sizes of microemulsion region for each phase diagram were compared to identify optimum composition of microemulsion. Larger microemulsion region refer to greater self-microemulsification efficiency of the tested ternary formulation. When Transcutol HP was used in equal ratio of Cremophore EL (Figure 5A Smix 1:1), there existed a small, easily flowable microemulsion zone. This is described by the phenomena that cosurfactant may allow the oil phase to enter the surfactant’s hydrophobic region. This will lead to an additional reduction in interfacial tension and hence increase the fluidity of the system. Increasing the amount of Cremophore EL in relation to Transcutol HP (Smix 2:1 and 3:1) led to a considerable increase in microemulsion region (Figure 5B,C). Further increase in Cremophore EL concentration (Smix 4:1) did not increase in the micro-emulsification region, but rather, it showed a little reduction in micro-emulsification area (Figure 5D). This indicated the accomplishment of optimum emulsification when Smix is at 3:1. Therefore, among all the Smix ratios studied, a 3:1 ratio of Smix was selected as optimal due to maximum microemulsification efficiency [19,20].

Additionally, it is evident from ternary plots that adding more Cremophore EL to Smix increased the resulting emulsion’s visual clarity. This can be explained by the fact that raising the surfactant concentration at interface may ultimately cause a reduction in oil content. This may lead to a reduced droplet size of generated microemulsion resulting in a more and more clear emulsion.

### 3.5. Experimental Optimization

#### 3.5.1. Selection of Formulations

Smix ratio of 3:1 for Cremophore EL and Transcutol HP yielded maximum self microemulsification efficiency. Hence, it was selected for further study. However, for further experimental optimization, entire range of compositions in self-micro-emulsifying region of ternary plot for Smix ratio of 3:1 was selected.

#### 3.5.2. Design of Experiment

Optimization of SMEDDS was carried out using scheffe mixture design (Table 1). Design Expert 13 software was used for suggesting appropriate fitting models and relationship between different variables using outcomes of various responses (Table 2). In the present study, drug loading, droplet size, emulsification time, and transmittance were selected as essential variables for optimization of a stable self-emulsifying microemulsion. A high drug loading capacity of microemulsion allows maximum drug to be encapsulated in SEDDS allowing greater drug to reach site of action. A smaller droplet dimensions present greater surface area of micelles for intestinal membrane interactions enabling enhanced drug absorption and rapid drug dissolution. A short emulsification time is a good indicator of self-emulsifying properties of microemulsion and also attributes to the non-existence of gel phase formation during dilution of microemulsion. Transmittance was selected to optimize a transparent homogenous SEDDS when dispersed in continuous aqueous medium [21].

The outcomes of responses $Y_{1}$ and $Y_{4}$ were fitted to linear statistical model whereas $Y_{2}$ and $Y_{3}$ were fitted to quadratic model, respectively. Model *p* value, $R^{2}$, adjusted $R^{2}$, and adequate precision values were statistical parameters considered while evaluation of each fitted statistical model. The *p* value less than 0.05 for each model suggested that the model terms are significant at 95% CI. The variability of response outcomes reflected by each model were estimated by $R^{2}$ and adjusted $R^{2}$. All values of $R^{2}$ and adjusted $R^{2}$ greater than 0.9 for $Y_{2}$, $Y_{3}$, and $Y_{4}$ pointed out that experimental data was in reasonable agreement to the fitted values. Even though the $R^{2}$ and adjusted $R^{2}$ value for $Y_{1}$ are 0.9398 and 0.8946, still adequate precision of 12.3888 (i.e., adequate precision greater than 4) indicates that the statistical model could be adequately utilized to navigate design space. All statistical models had a similar $R^{2}$ and adjusted $R^{2}$ values (i.e., the difference is less than 0.2 between $R^{2}$ and adjusted $R^{2}$) which suggested goodness-of-fit of all models [22,23].

#### 3.5.3. Optimization of Variables

**Figure 6:**
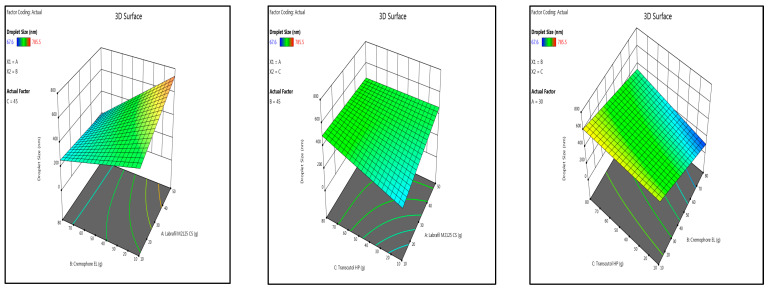

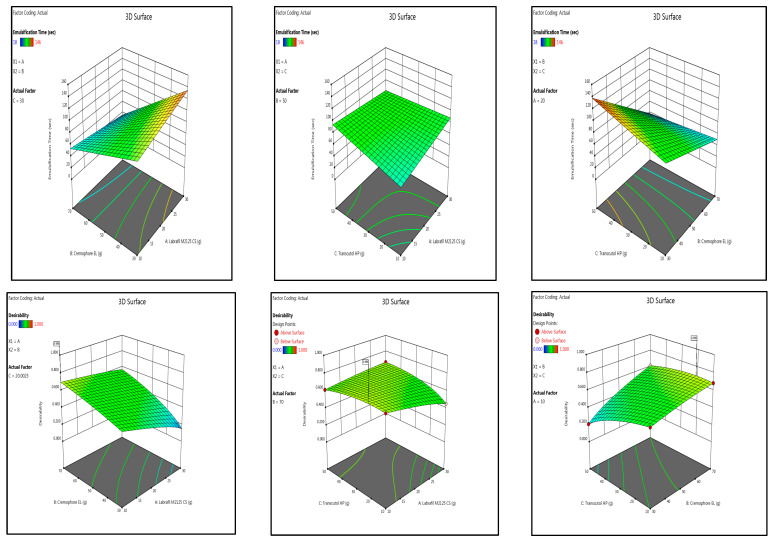
Three-dimensional surface plot of droplet size, emulsification time, and Desirability plot of optimized SMEDDS.

A stable microemulsion possess desired characteristics as mentioned in Table 2. In this present study, desirability function was utilized to optimize all the variables by considering various response outcomes. The objective of optimization was set at maximum values of $Y_{1}$ and $Y_{4}$ and minimum values for $Y_{2}$ and $Y_{3}$. Desirability plot showing the influence of three variables on the selected responses was displayed in Figure 6.

### 3.6. In Vitro Enzymatic Degradation Studies

#### Stability Assessment against Proteolytic Degradation

The enzymatic barrier of GIT caused by a highly intensive proteolytic enzyme degradation is one of the major barriers in improving the oral bioavailability of insulin [24]. As a result, a SEDDS was formulated to prevent the enzymatic breakdown of intestine in the intestine. Thus, a stability study was conducted to evaluate the stability of Ins-SDS and HIP loaded optimized SMEDDS against proteolytic degradation as compared to native insulin.

**Figure 7:**
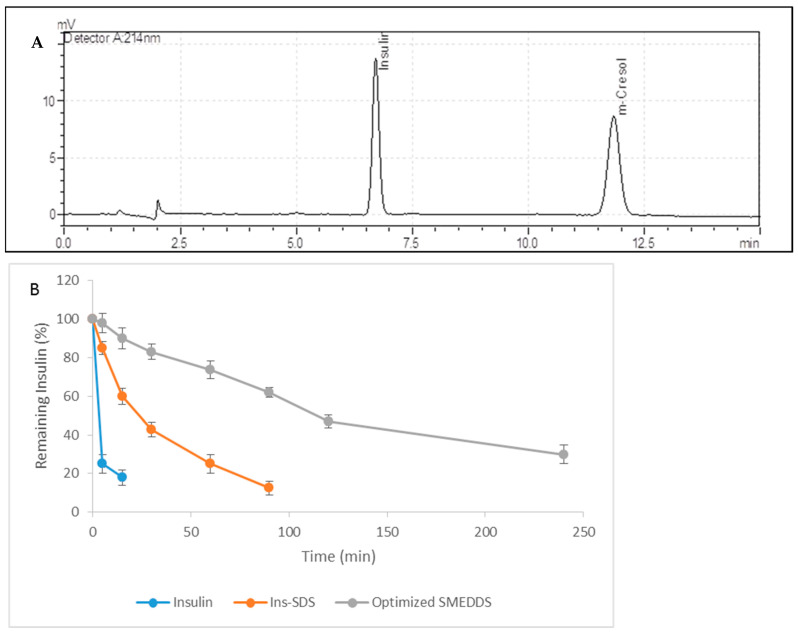
(**A**): Quantification of Insulin by HPLC, (**B**): Stability Profile of insulin, Ins-SDS HIP complex, and optimized SMEDDS against proteolytic degradation.

Figure 7B represents the degradation profile of Insulin, Ins-SDS, and optimized SMEDDS in SIF as a function of time. As expected, the native insulin was rapidly degraded within the first 15 min, indicating that insulin is highly unstable in GI tract. Ins-SDS complex was also metabolized completely but this degradation was much slower as compared to free insulin. This suggests that HIP improved the resistance of insulin against enzymatic degradation.

However, the optimized SMEDDS displayed considerable resistance against enzymatic degradation in SIF. In total, 47% of insulin loaded in optimized SMEDDS in the form of Ins-SDS complex remained stable after 2 h. This might be attributed to the fact that Ins-SDS complex was embedded in the oil droplets of microemulsion, whereas hydrophilic nature of GI enzymes does not allow them to penetrate hydrophobic region of microemulsion protecting the insulin from enzymatic degradation [15]. On the other hand, this shielding effect is confined until Ins-SDS remains embedded in oil droplets or SMEDDS does not degrade. As bile salts and lipase in intestine are able to digest lipid phase of microemulsion, exposing the Ins-SDS complex to enzymatic degradation. However, compared to Insulin and Ins-SDS, the optimized SMEDDS demonstrated higher effectiveness in preventing Ins-SDS from enzymatic degradation increasing the oral bioavailability of insulin.

### 3.7. In Vivo Study

#### Antidiabetic Effect Study in Diabetic Rats

**Figure 8:**
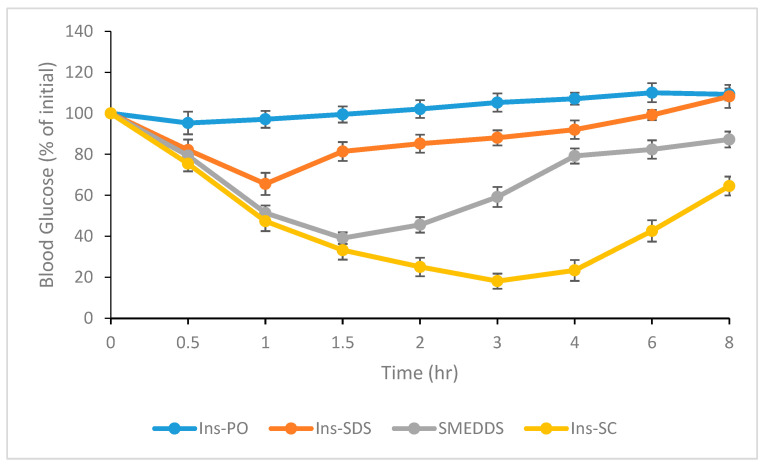
Antidiabetic activity study in diabetic rats after administration of Ins-PO (50 IU/kg), Ins-SDS (50 IU/kg), optimized SMEDDS (50 IU/kg), and Ins-SC (5 IU/kg).

In the present study all characterizations revealed that hydrophobically ion paired insulin with SDS (referred as HIP) maintained its structural integrity. Additionally, the bioactivity of orally administered HIP insulin was assessed directly by in vivo study in diabetic rats and compared with free insulin administered by both oral and subcutaneous route. Figure 8 displayed the hypoglycemic effect induced by (i) oral administration of pure insulin (Ins-PO), (ii) Ins-SDS complex alone, (iii) optimized SMEDDS formulation loaded with HIP, and (iv) insulin given subcutaneously.

Maximum reduction in blood glucose level was induced by insulin administered subcutaneously, and this effect lasted for the entire duration of the experiment, i.e., up to 8 h after the administration or start of treatment. In contrast, orally administered insulin solution induced negligible anti-diabetic effect. In the present study a separate group of rats was also administered with phosphate buffer saline (PBS). However, the observed results were not significantly different with the groups administered with insulin solution. Therefore, such data for PBS group is not presented in Figure 8.

Ins-SDS complex displayed slight reduction in glycemic level in first 1 h. This effect persisted for up to 2 h after oral administration. Later on, blood glucose concentration returned slowly to initial levels during 8 h. However, optimized SMEDDS loaded with HIP showed marked reduction in blood glucose level up to eight hours of study. Therefore, in the present study the observation study period was constrained up to 8 h instead of 24 h. These results suggests that present optimized SMEDDS loaded with HIP have the potential to improve the oral bioavailability of insulin compared to Ins-SDS complex alone and show marked hypoglycemic effect in SDS induced diabetic rats up to eight hours.

## 4. Conclusions

An Ins-SDS loaded SMEDDS was effectively optimized using Two factorial design. Three independent variables—$X_{1}$ (Labraphil M2125 CS), $X_{2}$ (Cremophore EL), and $X_{3}$ (Transcutol HP) were optimized at 10%, 70%, and 20%, respectively, resulting in drug loading (0.802%), droplet dimensions (249.5 nm), emulsification time (51.3 s), and transmittance (86.46%) with <10% prediction error. The optimized SMEDDS displayed considerably enhanced resistance to proteolytic degradation as compared to native insulin and Ins-SDS complex. This shows the potential of SMEDDS in improving oral bioavailability of macromoleuclar proteins. However, this present study utilized a STZ induced diabetic rat model; further evaluation of more sophisticated model using peptidase inhibitors simultaneously may provide better insights for clinical translation of SMEDDS into commercially viable products in the future.

## Figures and Tables

**Table 1:** Scheffe’s mixture design, Independent and Dependent variables.

Independent Variables	Range	
	Minimum (*w*/*w* %)	Maximum (*w*/*w* %)
$X_{1}$: Labrafil M2125 CS	10	30
$X_{2}$: Cremophore EL	30	70
$X_{3}$: Transcutol HP	10	50
**Dependent Variables**	**Goals**	
$Y_{1}:$ Drug Loading (wt%)	Maximum	
$Y_{2}$: Droplet Size (nm)	Minimum	
$Y_{3}$: Emulsification Time (s)	Minimum	
$Y_{4}$: Transmittance (%)	Maximum	

**Table 2:** Model fitting and statistical analysis summary.

Responses	Suggested Model	Model *p*-Value	$R^{2}$	$\mathrm{Adjusted}\;R^{2}$	Adequate Precision
$Y_{1}$: Drug Loading	Linear	0.0067	0.9398	0.8946	12.3888
$Y_{2}$: Droplet Size	Quadratic	0.0252	0.9998	0.9987	84.7940
$Y_{3}$: Emulsification Time	Quadratic	0.0381	0.9996	0.9971	54.5349
$Y_{4}$: Transmittance	Linear	0.003	0.9871	0.9774	26.2299

## Data Availability

All the data are contained in the manuscript.
